# Tocilizumab, netakimab, and baricitinib in patients with mild-to-moderate COVID-19: An observational study

**DOI:** 10.1371/journal.pone.0273340

**Published:** 2022-08-24

**Authors:** Ekaterina A. Bryushkova, Valeria D. Skatova, Zinaida Y. Mutovina, Alena I. Zagrebneva, Daria S. Fomina, Tatyana S. Kruglova, Anna A. Akopyan, Irina D. Strazhesko, Sergey A. Lukyanov, Olga N. Tkacheva, Maryana A. Lysenko, Dmitry M. Chudakov

**Affiliations:** 1 Pirogov Russian National Research Medical University, Moscow, Russia; 2 Shemyakin and Ovchinnikov Institute of Bioorganic Chemistry of the Russian Academy of Sciences, Moscow, Russia; 3 Lomonosov Moscow State University, Moscow, Russia; 4 City Clinical Hospital No.52 of Moscow Healthcare Department, Moscow, Russia; 5 I.M. Sechenov First Moscow State Medical University of the Ministry of Health of the Russian Federation, Moscow, Russian Federation; University of Hail, SAUDI ARABIA

## Abstract

**Objective:**

The aim of the study was to assess inflammatory markers and clinical outcomes in adult patients admitted to hospital with mild-to-moderate COVID-19 and treated with a combination of standard-of-care (SOC) and targeted immunosuppressive therapy including anti-IL-17A (netakimab), anti-IL-6R (tocilizumab), or JAK1/JAK2 inhibitor (baricitinib) or with a standard-of-care therapy alone.

**Methods:**

The observational cohort study included 154 adults hospitalized between February and August, 2020 with RT-PCR-confirmed SARS-CoV-2 with National Early Warning Score2 (NEWS2) < 7 and C-reactive protein (CRP) levels ≤ 140 mg/L on the day of the start of the therapy or observation. Patients were divided into the following groups: I) 4 mg baricitinib, 1 or 2 times a day for an average of 5 days (n = 38); II) 120 mg netakimab, one dose (n = 48); III) 400 mg tocilizumab, one dose (n = 34), IV) SOC only: hydroxychloroquine, antiviral, antibacterial, anticoagulant, and dexamethasone (n = 34).

**Results:**

CRP levels significantly decreased after 72 h in the tocilizumab (p = 1 x 10^-5^) and netakimab (p = 8 x 10^-4^) groups and remained low after 120 h. The effect was stronger with tocilizumab compared to other groups (p = 0.028). A significant decrease in lactate dehydrogenase (LDH) levels was observed 72 h after netakimab therapy (p = 0.029). NEWS2 scores significantly improved 72 h after tocilizumab (p = 6.8 x 10^-5^) and netakimab (p = 0.01) therapy, and 120 h after the start of tocilizumab (p = 8.6 x 10^-5^), netakimab (p = 0.001), or baricitinib (p = 4.6 x 10^-4^) therapy, but not in the SOC group. Blood neutrophil counts (p = 6.4 x 10^-4^) and neutrophil-to-lymphocyte ratios (p = 0.006) significantly increased 72 h after netakimab therapy and remained high after 120 h. The percentage of patients discharged 5-7 days after the start of therapy was higher in the tocilizumab (44.1%) and netakimab (41.7%) groups than in the baricitinib (31.6%) and SOC (23.5%) groups. Compared to SOC (3 of the 34; 8.8%), mortality was lower in netakimab (0 of the 48; 0%, RR = 0.1 (95% CI: 0.0054 to 1.91)), tocilizumab (0 of the 34; 0%, RR = 0.14 (95% CI: 0.0077 to 2.67)), and baricitinib (1 of the 38; 2.6%, RR = 0.3 (95% CI: 0.033 to 2.73)) groups.

**Conclusion:**

In hospitalized patients with mild-to-moderate COVID-19, the combination of SOC with anti-IL-17A or anti-IL-6R therapy were superior or comparable to the combination with JAK1/JAK2 inhibitor, and all three were superior to SOC alone. Whereas previous studies did not demonstrate significant benefit of anti-IL-17A therapy for severe COVID-19, our data suggest that such therapy could be a rational choice for mild-to-moderate disease, considering the generally high safety profile of IL-17A blockers. The significant increase in blood neutrophil count in the netakimab group may reflect efflux of neutrophils from inflamed tissues. We therefore hypothesize that neutrophil count and neutrophil-to-lymphocyte ratio could serve as markers of therapeutic efficiency for IL-17A-blocking antibodies in the context of active inflammation.

## Introduction

SARS-CoV-2 remains a pandemic pathogen with a high fatality rate, and efficient clinical protocols are still under development. After initial viral replication, the next and often most devastating stage of disease is driven by excessive or mistargeted host inflammatory response. Severely ill patients exhibit increased blood levels of interleukin (IL)-6, IL-1*β*, IL-2, IL-8, and IL-17, tumor necrosis factor *α* (TNF-*α*), granulocyte colony-stimulating factor (G-CSF), and interferon (IFN)-*γ* [1–5]. Increased IL-6 correlates with increased C-reactive protein (CRP) and both are elevated in non-survivors relative to survivors [6, 7]. As such, the high potential of immunosuppressive therapies currently applied for rheumatologic disorders ranging from dexamethasone and glucocorticosteroids to JAK inhibitors and selective cytokine inhibitors as a treatment option for COVID-19 is now widely accepted [8–13].

This study compares three therapies affecting linked inflammatory pathways.

Tocilizumab [14] is an anti-IL-6R monoclonal antibody that blocks IL-6 signaling and trans-signaling [15]. This suppresses the pleiotropic effects of IL-6, which include activation of acute-phase protein production, promotion of Th17 and Th2 helper T cells, and suppression of Th1 helper T cell differentiation [16]. Tocilizumab has previously been used to treat severe rheumatological diseases and cytokine release syndrome in the context of chimeric antigen receptor (CAR) T cell therapy [17, 18]. For this latter reason, many clinical centers have initially opted to apply tocilizumab to block cytokine storm induced by severe COVID-19 [11, 13, 19, 20]; currently, such treatment is recommended for hospitalized patients who are receiving systemic corticosteroids and require supplemental oxygen, non-invasive or invasive mechanical ventilation, or extracorporeal membrane oxygenation [21].

Netakimab is an anti-IL-17A monoclonal antibody recommended for treatment of moderate-to-severe plaque psoriasis [22], ankylosing spondylitis [23] (ClinicalTrials.gov: NCT03447704) and psoriatic arthritis [24] (ClinicalTrials.gov: NCT03598751). IL-17, which is produced by Th17, Tc17, and other clonal type 3-programmed ROR*γ*t-positive lymphoid cells [25], triggers production of pro-inflammatory cytokines including granulopoiesis-inducing G-CSF, the systemic inflammatory cytokines IL-6, IL-1*β*, and TNF*α*, neutrophil-attracting IL-8, as well as tissue-disintegrating matrix metalloproteinases [26]. IL-6 in turn promotes the differentiation of Th17 cells, resulting in a positive feedback loop [27]. There is substantial evidence that infection by coronaviruses, including MERS-CoV and SARS-CoV-2, directs the immune response along the Th17 pathway [28–32], and IL-17 may represent one of the key mediators of tissue damage, acute respiratory distress syndrome (ARDS), and cytokine storm in such viral infections [32–39].

Baricitinib is a small molecule JAK1/JAK2 inhibitor used for the treatment of rheumatoid arthritis. Baricitinib affects intracellular signaling by decreasing the level of phosphorylated STAT proteins, thereby blocking the proinflammatory signals IL-6, IL-12, IL-23 and IFN-*γ* [40]. Baricitinib exerts a variety of immunosuppressive effects, including suppression of the differentiation of Th1 and Th17 cells [41]. This outcome links its mode of action with anti-IL-17A and anti-IL-6/IL-6R monoclonal antibodies. Based on positive clinical data [42–44], baricitinib has been approved for COVID-19 treatment by the European Commission and FDA. The aim of this study was to assess inflammatory markers and clinical outcomes in hospitalized, mild-to-moderate COVID-19 patients treated with the combination of one of the three agents: netakimab, tocilizumab, and baricitinib with standard of care (SOC) therapy or with SOC only without targeted immunosuppression.

## Methods

### Study design and participants

This observational cohort study was conducted in COVID-19 care units at two tertiary hospitals in Moscow (City Clinical Hospital No. 52 of the Moscow Healthcare Department, and the Russian Gerontology Research and Clinical Center at Pirogov Russian National Research Medical University) on patients with mild or moderate COVID-19 pneumonia. Both centers contributed data on baricitinib, netakimab, tocilizumab, and SOC treatment. The study participants were adults with RT-PCR-confirmed SARS-CoV-2 infection, who were admitted to the hospital between February and August 2020. Eligible patients had symptoms of acute respiratory infection (fever, muscle pain, cough) and radiologically-defined viral pneumonia as assessed by computed tomography.

Patients were excluded if their National Early Warning Score 2 (NEWS2) metric—which falls on a scale of 0 to 20 based on physiological variables including blood pressure, heart rate, respiratory rate, temperature, oxygen saturation, and level of consciousness [45–47]—was unknown or higher than 6 points at the start therapy day. Patients were also excluded if their serum CRP level at the start therapy day was higher than 140 mg/L, if there was no information about death or hospital discharge, if they were receiving combination or non-standard regimens of the investigated therapies, if targeted immunosuppression therapy started later than 72 hours after admission to the hospital, or if they had severe hematological, renal, or liver function impairment or evidence of concomitant bacterial infections. All patients who received tocilizumab, netakimab, or baricitinib provided written informed consent. The study was approved by the Local Ethical Committee of the City Clinical Hospital No.52.

### Procedures

All patients received SOC treatment at the time of hospital admission according to the corresponding COVID-19 Russian guidelines 2020 [48].

Despite the situational variability of SOC treatment for different patients, it basically included symptomatic treatment (paracetamol for fever relief; moisturizers/elimination drugs, nasal decongestants for complex therapy of rhinitis and/or nasopharyngitis and mucoactive, bronchodilator and others for complex therapy of bronchitis) and etiotropic treatment: hydroxychloroquine (HCQ, 400 mg twice on day 1, followed by 200 mg twice per day on days 5–10), azithromycin (500 mg once per day for 5 days), lopinavir–ritonavir (400/100 mg twice per day for 14 days), and low molecular-weight heparin, occasionally supported by dexamethasone treatment or convalescent plasma transfusion according to indications and contraindications.

In addition to SOC treatment, patients also received tocilizumab or baricitinib or netakimab treatment. Netakimab was administered by a single subcutaneous injection of 120 mg, tocilizumab was administered by a single intravenous dose of 400 mg, and baricitinib was administered at a dose of 4 mg once or twice per day for 3–8 days (5 days on average).

A prior randomization of patients before the targeted immunosuppressive therapy prescription was not carried out. The treatment plan for each patient in the inpatient department was approved by the attending physician based on the severity of the disease and results of laboratory and instrumental studies. To reduce the potential biases, we used our own inclusion and exclusion criteria listed above in Study design.

### Outcomes

The primary outcomes of the study were the biochemical inflammatory markers CRP (mg/L) and lactic dehydrogenase (LDH, U/L), absolute neutrophil count (ANC) and absolute lymphocyte count (ALC) in blood (x 10^9^/L), and the neutrophil-to-lymphocyte ratio (NLR). The level of C-reactive protein (mg/L) was measured by quantitative immunoturbidimetric assay (CRP System reagent kit for HUMASTAR 600; Human GmbH, Germany); blood lactate dehydrogenase (LDH, U/L) was estimate by enzymatic UV-Kinetic method (LDH SCE mod.liquiUV kit for HUMASTAR 600; Human GmbH, Germany), an absolute lymphocyte (ALC) and neutrophil (ANC) count was assessed by using the Auto 5-Diff Hematology Analyzer BC-6800 (Mindray Global; Shenzhen Mindray Bio-Medical Electronics Co., Ltd).

Secondary outcomes were the NEWS2 score, time until discharge from the hospital, and mortality rate. For each patient all parameters were estimated at baseline (day 0), 72 h after initiation of treatment, and 120 h after initiation of treatment.

### Statistical analysis

Continuous variables were expressed as median (IQR) and compared by Kruskal Wallis test. At baseline (0 h), we assessed the similarity of NEWS2, LDH, and CRP distribution in the four cohorts using the Kruskal-Wallis and Dunn test. For paired comparisons between two time points, we used the Wilcoxon test. P-values ≤ 0.05 were considered significant, all p-values are shown with adjustment for multiple testing using Bonferroni correction. Analyses were done by R version 1.2.5. In order to extract maximal statistical information, we independently assessed the individual dynamics of parameters between baseline and 72 h (Δ0–72) and between baseline and 120 h (Δ0–120), including all patients for which the parameter of interest was known at both analyzed time points. As such, the number of patients included in each analysis differs. Additionally, the individual dynamics of CRP, LDH, NEWS2 score, and blood cell counts was also assessed for patients for whom the results of sequential measurements were available for all three timepoints. Again, the number of patients included in each analysis differed.

## Results

### Baseline patient characteristics

A total of 154 patients with mild-to-moderate COVID19 were included in the study to receive the combination of one of the three agents: baricitinib (baricitinib group, n = 38), netakimab (netakimab group, n = 48), tocilizumab (tocilizumab group, n = 34) with SOC therapy, or SOC therapy only (SOC group, n = 34) without targeted immunosuppression. Baseline characteristics of patients in the compared groups are summarized in Tables 1 and 2.

**Table 1:** General patient characteristics.

Parameter		Baricitinib (n = 38)	Netakimab (n = 48)	Tocilizumab (n = 34)	SOC (n = 34)
Age (in years) mean [SD]		60.7 [12.8]	63.4 [12.7]	60.2 [8.7]	66.1 [13.4]
Sex, % (n)	male	42 (16)	48 (23)	47 (16)	32 (11)
	female	58 (22)	52 (25)	53 (18)	68 (23)
Days in hospital before start of the therapy, % (n)	0	34 (13)	58 (28)	82 (28)	-
	1	55 (21)	38 (18)	18 (6)	
	2	11 (4)	4 (2)	0 (0)	
NEWS2, % (n)	0–4	82 (31)	92 (44)	85 (29)	79 (27)
	5–6	18 (7)	8 (5)	15 (5)	21 (7)
Hydroxychloroquine treatment, % (n)	yes	68 (26)	10 (5)	35 (12)	91 (31)
	no	32 (12)	90 (43)	65 (22)	9 (3)

Data are mean [SD] or % (n), where n = number of patients; % = percentage; SD = standard deviations; NEWS2 = National Early Warning Score 2.

**Table 2:** Baseline clinical characteristics of patients.

Parameter	Baricitinib (n = 38)	Netakimab (n = 48)	Tocilizumab (n = 34)	SOC (n = 34)	p-value
NEWS2, median (IQR)	2 (1–3)	2 (1–3)	3 (2–4)	2 (1–3.75)	0.007
ANC, median (IQR)	5.05 (2.8–7.06)	3.05 (1.92–4.95)	3.25 (2.22–4.47)	4.2 (2.85–6.47)	> 0.05
ALC, median (IQR)	1.65 (1.08–2.17)	1 (0.72–1.6)	1 (0.62–1.37)	1.2 (0.87–1.52)	> 0.05
NLR, median (IQR)	2.42 (1.65–4.11)	2.32 (1.77–5.46)	3.94 (2.36–6.53)	3.3 (1.95–5.65)	0.011
CRP, median (IQR)	28 (13.28–57.65)	17.34 (8.32–52.36)	27.39 (14.09–50.44)	27.05 (14.98–89.91)	> 0.05
LDH, median (IQR)	327 (220.4–491.5)	247.6 (209.4–298.8)	286 (272.6–371.1)	282.2 (216.4–350.4)	0.039

Data presented as median (IQR), where IQR = interquartile range; NEWS2 = National Early Warning Score 2; ANC = absolute neutrophil count (x 10^9^/L); ALC = absolute lymphocyte count (x 10^9^/L); NLR = neutrophil-to-lymphocyte ratio; CRP = C-reactive protein (mg/L); LDH = lactic dehydrogenase (U/L); n = number of patients; IQR = interquartile range. Evenness of parameters distribution between groups was examined with Kruskal-Wallis and Dunn test. Paired comparisons between groups are shown in S1 Fig.

Although the numbers of mild and moderate patients were roughly comparable in all groups, the Kruskal-Wallis and Dunn tests showed a significant difference in initial NEWS2 scores for tocilizumab (median = 3) compared to the netakimab group, while there were no significant differences between the baricitinib, netakimab, and SOC groups. Differences in LDH levels and NLR between groups were also significant at the start of the therapy (p = 0.04 and 0.012, respectively; Table 2, S1 Fig). According to the approved regimens (corresponding COVID-19 Russian guidelines 2020), baricitinib was recommended for the treatment of patients with mild to moderate COVID-19 in combination with HCQ (unless prolongation of the QT interval on the electrocardiogram was detected at the time of appointment, which was considered a contraindication to the use of HCQ). Therefore, in the baricitinib + SOC group there was an increased proportion of patients receiving HCQ (68%).

Despite the previously reported potential antiviral and anti-inflammatory effects of HCQ, large clinical research based on the five blinded, placebo-controlled RCTs enrolled a total of 1138 individuals showed no clinical benefit on HCQ use as treatment for hospitalized patients with COVID-19 [49]. At the same time, there was also no evidence that the use of HCQ delays recovery or worsens the clinical parameters of COVID-19 patients. The effect of HCQ on important outcomes, including time to symptom resolution, admission to hospital, and duration of mechanical ventilation, remains uncertain (WHO Therapeutics and COVID-19: living guideline).

Therefore, we believe that the increased proportion of HCQ-treated patients in the baricitinib group did not essentially affect the observed results.

### CRP and LDH

**Fig 1:**
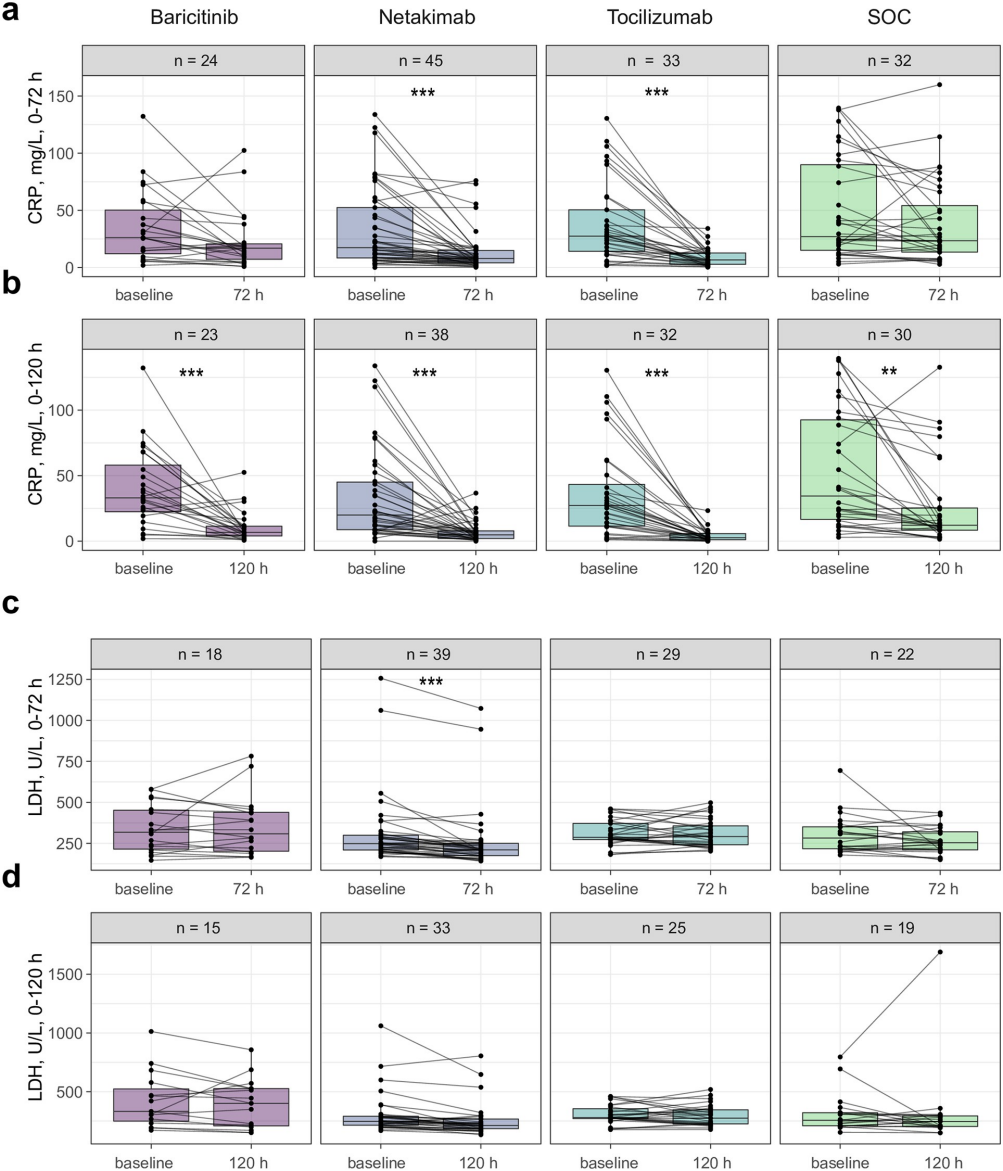
Changes in CRP and LDH levels over time. Measured levels of C-reactive protein (CRP) at baseline versus 72 h (**a**) or 120 h (**b**). Measured levels of lactate dehydrogenase (LDH) at baseline versus 72 h (**c**), or 120 h (**d**). Dots show measurements for individual patients, n = number of patients with available values of the corresponding parameters. Wilcoxon test: **p < 0.01; ***p< 0.001.

We independently assessed the individual dynamics of CRP and LDH for two separate datasets: patients with known parameters at baseline and 72 h (Δ0–72) and baseline and 120 h (Δ0–120) S1 Table. This allowed us to avoid excluding patients from the analysis who lacked CRP or LDH data at one of the time-points. CRP levels significantly decreased after 72 h in the tocilizumab (p = 1 x 10^-5^) and netakimab (p = 8 x 10^-4^) groups, but not in the baricitinib (p > 0.05) and SOC (p > 0.05) groups (Fig 1a). The amplitude of this effect was higher in the tocilizumab group compared to the other groups (p = 0.028, S2 Fig). After 120 h, CRP levels significantly decreased in all groups, but remained high for some individual patients in the SOC group (Fig 1b). We only observed significant reduction of LDH levels in the netakimab group after 72 h (p = 0.029, Fig 1c and 1d). The extent of LDH reduction did not significantly differ between groups after either 72 or 120 h (p > 0.05, S2 Fig).

We also assessed the individual dynamics of CRP and LDH for those patients for whom sequential measurements were available for all three time-points. For the vast majority of patients in the netakimab and tocilizumab groups, CRP decreased over time, while more heterogeneous dynamics were observed in the baricitinib and SOC groups (S3 Fig).

### NEWS2 and blood cell counts

**Fig 2:**
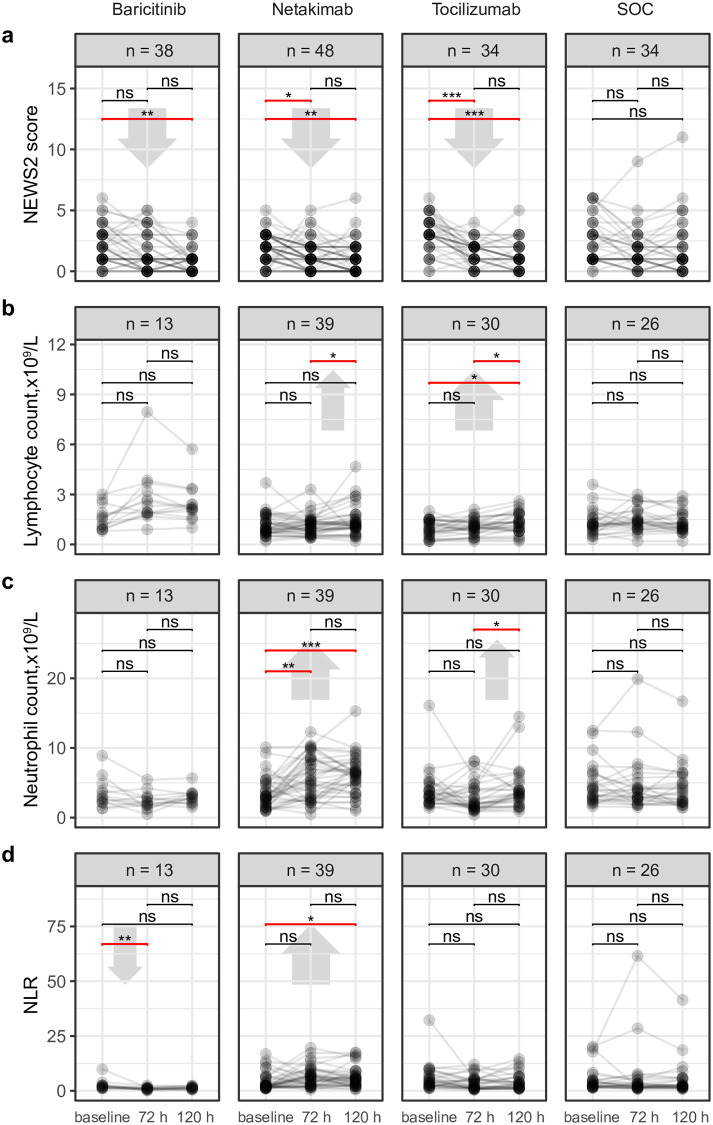
Individual dynamics of blood parameters. **a**—NEWS2 score; **b**—absolute lymphocyte count; **c**—absolute neutrophil count; **d**—neutrophil-to-lymphocyte ratio (NLR); n = number of patients with available values of the corresponding parameters. Wilcoxon test: *p < 0.05; **p < 0.01; ***p < 0.001; ns = non-significant.

NEWS2 scores and blood cell counts were available from all time-points for most patients (Fig 2, S2 Table). In this analysis, NEWS2 scores significantly improved 72 h after tocilizumab and netakimab therapy, and 120 h after the start of tocilizumab, netakimab, or baricitinib therapy, but not in the SOC group. Absolute neutrophil count (ANC) increased significantly and prominently 72 h after netakimab therapy and remained high after 120 h. Blood neutrophil-to-lymphocyte ratio (NLR) significantly decreased after 72 h in the baricitinib group, but increased 120 h after netakimab therapy.

We also performed separate analysis of NEWS2 score and blood cell counts for patients with known parameters at baseline and 72 h (Δ0–72) and baseline and 120 h (Δ0–120), which allowed us to include more patients (S1 Table). This analysis confirmed the increase of ANC and NLR in the netakimab group at both 72 h and 120 h, as well as the decrease of NLR in the baricitinib group.

### Day of discharge and mortality data

Most patients were discharged from the hospital by the 14th day after initiation of therapy (Table 3), without significant differences between the groups (p > 0.05).

**Table 3:** The outcome timepoints and the mortality level of patients.

Group		Baricitinib (n = 38)	Netakimab (n = 48)	Tocilizumab (n = 34)	SOC (n = 34)
Outcome, % (n)	hospital discharge	97 (37)	100 (48)	100 (34)	91 (31)
	death	3 (1)	0 (0)	0 (0)	8.8 (3)
Days in hospital after starting therapy, % (n)	≤ 4	2.6 (1)	4.2 (2)	0 (0)	0 (0)
	5–7	28.9 (11)	37.5 (18)	44.1 (15)	23.5 (8)
	8–14	57.9 (22)	47.9 (23)	47.1 (16)	70.6 (24)
	≥ 15	10.5 (4)	10.4 (5)	8.8 (3)	5.9 (2)

Data presented as % (n), where n = number of patients; % = percentage. Evenness of parameters distribution between groups was examined with Kruskal-Wallis and Dunn test.

The percentage of patients who were discharged 5–7 days after the start of therapy was higher in the tocilizumab (44.1% of patients) and netakimab (41.7%) groups relative to the baricitinib (31.6%) and SOC (23.5%) groups. Mortality was higher in the SOC (8.8%) and baricitinib (3%) groups than in tocilizumab (0%) and netakimab (0%) groups (Table 3).

## Discussion

*Tocilizumab* was recommended on 6 July 2021 by the WHO for treatment of severe COVID-19. It was the second drug found to be effective against COVID-19 after dexamethasone, in September 2020 [50]. Some previous data have suggested that tocilizumab might reduce the risk of invasive mechanical ventilation or death in patients with severe [19] or moderate-to-severe [51] COVID-19 pneumonia. However, these results were controversial, because other clinical trials found that tocilizumab was not effective for preventing intubation or death in moderately ill patients [52, 53] (ClinicalTrials.gov, NCT04356937). The results of the COVACTA phase 3 clinical trial (ClinicalTrials.gov, NCT04320615) were published in summer 2020. This randomized controlled trial was conducted at 62 hospitals in nine countries (Canada, Denmark, France, Germany, Italy, the Netherlands, Spain, the United Kingdom, and the United States) and included 438 patients. All patients were hospitalized with COVID-19 pneumonia and received an intravenous infusion of 8 mg/kg tocilizumab (800 mg maximum, n = 294) or placebo (n = 144). The main outcomes were clinical status at days 14 and 28, mortality at day 28, and number of ventilator-free days by day 28. Tocilizumab treatment did not yield significantly better clinical status or lower mortality than placebo at 28 days. Adverse events were similar and well balanced in the two trial groups, although incidence of infection was lower in the tocilizumab group [54].

The 11-month RECOVERY trial (ClinicalTrials.gov, NCT04381936) included 4116 patients who had hypoxia and evidence of inflammation (CRP ≥ 75 mg/L); 2022 patients received 400–800 mg tocilizumab plus SOC therapy, with 2094 patients only receiving SOC therapy. Almost all patients (82%) were also taking systemic steroids (e.g. dexamethasone). 28-day mortality was significantly lower in the tocilizumab group compared to the control group (31% vs 35%), along with a greater probability of discharge from hospital within 28 days (57% vs 50%) and lower risk of invasive mechanical ventilation or death (35% vs 42%) [13]. These results led to an update in WHO clinical guidelines and the inclusion of tocilizumab as a COVID-19 treatment.

Although the RECOVERY trial did not observe significant differences in the frequency of new cardiac arrhythmias between tocilizumab and control groups, and described only three reports of serious adverse reactions believed to be related to tocilizumab (otitis externa, *Staphylococcus aureus* bacteraemia, and lung abscess), many authors have mentioned significantly higher levels of other adverse effects of tocilizumab, including serious hepatic, pancreatic, and pulmonary reactions [55], cases of neutropenia, superinfections, reactivation of latent infections [56], and late-onset infections among patients receiving tocilizumab [57]. Nevertheless, the current consensus is that tocilizumab may be effective in severe COVID-19 disease, especially in patients with infection-related inflammation and lung damage [52]. In our study, tocilizumab produced a profound and stable decrease of CRP, and significant improvement in NEWS2 scores 72 h after therapy. The tocilizumab group had a higher percentage of patients discharged 5–7 days after the therapy, with lower mortality compared to the baricitinib and SOC, but not netakimab groups.

*Baricitinib.* At the start of this study (April 2020), there were no recommendations on the use of baricitinib for the treatment of COVID-19. The motivation for its use was to suppress pathological inflammatory response. Previous clinical trials on healthy volunteers have demonstrated that maximal inhibition of cytokine-induced STAT3 phosphorylation was observed 1–2 h after administration of baricitinib at single oral doses of 1, 5, or 10 mg, with levels returning to baseline by 16–24 h [58]. Previous clinical studies of baricitinib effectiveness and safety for patients with rheumatoid arthritis did not show any significant difference between 4 mg and 8 mg doses. Treatment-related adverse events were observed in 63% and 67% of patients receiving the 4 mg and 8 mg dosage, respectively; the incidence of serious cases (including infection) was 35% and 40%, respectively [59]. Based on these results, patients were maintained at a stable concentration of baricitinib, dosed at 4 mg one or two times a day for 3–8 days (five days on average). As a whole, studies of long-term (2–5.5 years) administration of baricitinib for rheumatoid arthritis have indicated good tolerability. The most common side effects in patients treated with 4 mg/day baricitinib include risk of pneumonia, herpes zoster, and infections of the urogenital tract, as well as deterioration of biochemical parameters, neutropenia, lymphopenia, anemia, thrombocytosis, and elevation of liver enzymes (ALT, AST), lipids, and creatine phosphokinase. Long-term use of baricitinib also increases the probability of developing deep venous thrombosis (DVT) and pulmonary embolism (PE) in patients belonging to corresponding risk groups. Baricitinib is therefore not recommended for patients with active tuberculosis or other infections, as well as patients with ALC < 0.5 × 10^9^/L, ANC < 1.0 × 10^9^/L or hemoglobin < 8 g/dL, and should be used with caution in patients with risk factors such as old age, obesity, history of DVT / PE, or use of selective COX-2 inhibitors [40]. There is no definitive answer as to whether the above-described side effects manifest after short-term (7–14 days) use of baricitinib for COVID-19. Retrospective studies at the University of Pisa (37 patients, 4 mg/day for 14 days) and Albacete Hospital (46 patients, 2 or 4 mg/day for 3–11 days) demonstrated that the beneficial effect of baricitinib became apparent at the fifth day of use and persisted until the end of observation (p < 0.0001). During that same time, transaminitis was observed in seven (19%) patients within 72 hours after starting baricitinib, and resulted in discontinuation of the drug in four cases; for the other three, the biochemical parameters stabilized on their own. In addition, five (14%) patients from Pisa got treatment-related infections. In the Albacete cohort, transaminitis was observed in 12 (26%) patients, but therapy was not terminated and the health indicators returned to normal regardless of the drug intake. Other side effects, probably associated with baricitinib treatment, were observed in nine (20%) patients [60].

More extensive results were obtained recently from a phase 3 double-blind, randomized placebo-controlled study (ClinicalTrials.gov, NCT04421027), which enrolled 1525 patients over 18 years old from 12 countries in Asia, Europe, North America, and South America with laboratory-confirmed COVID-19. Baricitinib or placebo was administered orally at a dose of 4 mg/day (or 2 mg/day for patients with baseline estimated glomerular filtration rate of 30–60 (mL/min)/1.73 m^2^) for 14 days or discharge, if it happened before 14 days. The baricitinib group achieved absolute risk reduction of five percentage points in all-cause mortality at 28 days, and 4.9 percentage points in all-cause mortality at 60 days. At the same time, the frequency of therapy-associated adverse effects was the same in the baricitinib and placebo groups. At least one treatment-related adverse event was observed for 45% of patients in the baricitinib group vs 44% in the placebo group, with serious adverse events described for 15% and 18% of participants, respectively. The combination of baricitinib with SOC did not lead to an increased risk of infections for this cohort of patients [43].

*Netakimab* is a humanized IgG1 monoclonal antibody against IL-17a that was registered in Russia in spring 2020 for the treatment of psoriasis [22], ankylosing spondylitis [23] (ClinicalTrials.gov: NCT03447704), and psoriatic arthritis (ClinicalTrials.gov: NCT03598751). Clinical trials suggest that netakimab is well tolerated. In a phase 3 clinical study of moderate-to-severe plaque psoriasis, PLANETA (ClinicalTrials.gov: NCT03390101) patients received netakimab 120 mg once every two or four weeks over the course of 12 weeks [22]. Treatment-emergent adverse events were registered in 9.4%, 10.7%, and 6.8% of patients receiving two-week, four-week, or placebo treatment, respectively (p > 0.05). All treatment-related adverse events were typical for IL-17 inhibitors: neutropenia, lymphopenia, and upper respiratory tract infections. The potential efficacy of netakimab for treating cytokine release syndrome in COVID-19 has been examined in at least two retrospective studies: in comparison with patients who did not receive anti-cytokine therapy or were treated by tocilizumab [61], and in comparison with SOC therapy including hydroxychloroquine, azithromycin, low-molecular-weight heparins, and corticosteroids [62]. Both studies indicated that netakimab is safe. The first study included COVID-19 patients with CRP > 60 mg/L, and the authors observed decreased CRP, fibrinogen, creatinine, and body temperature, and increased lymphocyte and platelet counts and alanine-transferase activity in all groups of patients 7–10 days after the start of therapy. The decrease in CRP levels was greater in the netakimab and tocilizumab groups than in the control group, and there was no difference between the netakimab and tocilizumab groups. Patients who received netakimab had a better survival rate among all three groups. In the second study, NEWS2 and CRP scores improved significantly on day three after the start of therapy for the netakimab group compared to the SOC group, but there was no difference in LDH levels and mortality [62]. Unlike in our study, patients with severe disease were included, with a median NEWS2 score of 7 for the whole cohort.

A study investigating hospitalized patients with severe COVID-19 treated with another IL-17 blocking antibody, secukinumab, showed that the therapy was well tolerated and was not associated with increase of adverse events or secondary infections compared to the SOC group. While no significant benefit was shown for the severe patients, a significant decrease of the number of thromboembolic events was demonstrated [63]. In our study of mild-to-moderate COVID19 patients, the netakimab group was the only one in which a significant decrease of both CRP and LDH parameters was observed 72 h after the start of therapy. This group was also characterized by a higher percentage of patients discharged 5–7 days after the therapy, as well as zero mortality.

It is remarkable that we observed a significant increase in peripheral neutrophil count specifically in the netakimab group (Fig 2), in the context of decreasing CRP (Fig 1a and 1b), LDH (Fig 1c and 1d), and NEWS2 (Fig 2) and a positive clinical outcome (Table 3). IL-17 triggers production of a range of pro-inflammatory cytokines and chemokines, including neutrophil-attracting IL-8 [26]; in ARDS patients with a systemic inflammatory response, alveolar levels of IL-17 are increased, and this is associated with organ dysfunction and increased alveolar neutrophils and alveolar permeability [34, 35]. Therefore, our interpretation of these results is that blockage of IL-17 activity could lead to the partial loss of neutrophil chemotaxis to the inflamed lung tissues, and their efflux from inflamed tissues results in the observed increase in peripheral blood neutrophil count. As such, the ANC and NLR parameters could paradoxically be considered as markers of therapeutic efficacy for IL-17-blocking antibodies in the context of active inflammation.

## Conclusion

We conclude that the use of any of the three targeted immunosuppression regimens under consideration: tocilizumab, netakimab, or baricitinib in combination with the standard of care therapy, results in an improvement in CRP levels and a positive trend in NEWS2 score 120 h after the start of the therapy in patients with mild to moderate coronavirus infection relative to SOC therapy alone. However, our results indicate that tocilizumab or netakimab might be more effective in alleviating inflammation within the first 72 hours of therapy. Potential adverse events should probably be the key criteria for selecting immunosuppressive therapy for such patients. Considering the generally high safety profile of anti-IL-17 blockers [64], those may represent the best option of the three for this category of patients. A prospective randomized trial will be necessary to verify our results, but we hope that our current observations will be useful in guiding therapy in individual cases based on the patient’s condition and potential risk of adverse events for each drug.

## Supporting information

S1 Fig Distribution of the NEWS2 score (A), CRP level (B), LDH level (C) and NLR (D) at baseline. Kruskal-Wallis and Dunn test, Bonferroni adjustment method: *p < 0.05; **p < 0.01; ***p < 0.001. Number of patients with available data are shown along the x-axis.
(PDF)

S2 Fig Changes in CRP and LDH levels 72 (A, C) or 120 hours (B, D) after the start of therapy. Kruskal-Wallis and Dunn test: *p < 0.05. Number of patients with available data are shown along the x-axis.
(PDF)

S3 Fig Individual dynamics of CRP (a) and LDH (b) across 120 h of treatment. Wilcoxon test: *p < 0.05; **p < 0.01; ***p < 0.001; ****p < 0.0001, ns = non-significant; n = number of patients with available data for all 3 time-points.
(PDF)

S1 Table Clinical characteristics of patients from the Δ0–72 h and Δ0–120 h groups. Data are median (IQR), where IQR = interquartile range; CRP = C-reactive protein (mg/L); LDH = lactic dehydrogenase (IU/L); ANC = absolute neutrophil count (x 10^9^/L); ALC = absolute lymphocyte count (x 10^9^/L); NLR = neutrophil-to-lymphocyte ratio; NEWS2 = National Early Warning Score 2; n = number of patients with corresponding data. P values were calculated using Kruskal-Wallis and Dunn test, Δ = p-value for Baricitinib group, ‡ = p-value for Netakimab group, # = p-value for Tocilizumab group, ⬛ = p-value for SOC group. P-values that do not significantly differ are shown as p > 0.05.
(PDF)

S2 Table Clinical characteristics of patients at three different timepoints. Data are median (IQR), where IQR = interquartile range; CRP = C-reactive protein (mg/L); LDH = lactic dehydrogenase (IU/L); ANC = absolute neutrophil count (x 10^9^/L); ALC = absolute lymphocyte count (x 10^9^/L); NLR = neutrophil-to-lymphocyte ratio; n = number of patients with data available.
(PDF)

S1 File Minimal dataset. (CSV)

## References

[1] McElvaney OJ, McEvoy NL, McElvaney OF, Carroll TP, Murphy MP, Dunlea DM, et al. Characterization of the Inflammatory Response to Severe COVID-19 Illness. Am J Respir Crit Care Med. 2020; 202(6):812–821. doi: 10.1164/rccm.202005-1583OC 32584597 PMC7491404

[2] Ghazavi A, Ganji A, Keshavarzian N, Rabiemajd S, Mosayebi G. Cytokine profile and disease severity in patients with COVID-19. Cytokine. 2021; 137:155323. doi: 10.1016/j.cyto.2020.155323 33045526 PMC7524708

[3] Leisman DE, Ronner L, Pinotti R, Taylor MD, Sinha P, Calfee CS, et al. Cytokine elevation in severe and critical COVID-19: a rapid systematic review, meta-analysis, and comparison with other inflammatory syndromes. Lancet Respir Med. 2020; 8(12):1233–1244. doi: 10.1016/S2213-2600(20)30404-5 33075298 PMC7567529

[4] Mehta P, McAuley DF, Brown M, Sanchez E, Tattersall RS, Manson JJ, et al. COVID-19: consider cytokine storm syndromes and immunosuppression. Lancet. 2020; 395(10229):1033–1034. doi: 10.1016/S0140-6736(20)30628-0 32192578 PMC7270045

[5] Yang L, Xie X, Tu Z, Fu J, Xu D, Zhou Y. The signal pathways and treatment of cytokine storm in COVID-19. Signal Transduct Target Ther. 2021; 6(1):255. doi: 10.1038/s41392-021-00679-0 34234112 PMC8261820

[6] Herold T, Jurinovic V, Arnreich C, Lipworth BJ, Hellmuth JC, von Bergwelt-Baildon M, et al. Elevated levels of IL-6 and CRP predict the need for mechanical ventilation in COVID-19. The Journal of allergy and clinical immunology. 2020; 146(1):128–136 e4. doi: 10.1016/j.jaci.2020.05.008 32425269 PMC7233239

[7] Santa Cruz A, Mendes-Frias A, Oliveira AI, Dias L, Matos AR, Carvalho A, et al. Interleukin-6 Is a Biomarker for the Development of Fatal Severe Acute Respiratory Syndrome Coronavirus 2 Pneumonia. Frontiers in immunology. 2021; 12:613422. doi: 10.3389/fimmu.2021.613422 33679753 PMC7930905

[8] Neubauer A, Johow J, Mack E, Burchert A, Meyn D, Kadlubiec A, et al. The janus-kinase inhibitor ruxolitinib in SARS-CoV-2 induced acute respiratory distress syndrome (ARDS). Leukemia. 2021; 35(10):2917–2923. doi: 10.1038/s41375-021-01374-3 34385593 PMC8358255

[9] Schett G, Manger B, Simon D, Caporali R. COVID-19 revisiting inflammatory pathways of arthritis. Nat Rev Rheumatol. 2020; 16(8):465–470. doi: 10.1038/s41584-020-0451-z 32561873 PMC7304381

[10] Les Bujanda I, Loureiro-Amigo J, Bastons FC, Guerra IE, Sanchez JA, Murgadella-Sancho A, et al. Treatment of COVID-19 pneumonia with glucocorticoids (CORTIVID): a structured summary of a study protocol for a randomised controlled trial. Trials. 2021; 22(1):43. doi: 10.1186/s13063-020-04999-4 33430891 PMC7797701

[11] Xu X, Han M, Li T, Sun W, Wang D, Fu B, et al. Effective treatment of severe COVID-19 patients with tocilizumab. Proc Natl Acad Sci U S A. 2020; 117(20):10970–10975. doi: 10.1073/pnas.2005615117 32350134 PMC7245089

[12] Cantini F, Goletti D, Petrone L, Najafi Fard S, Niccoli L, Foti R. Immune Therapy, or Antiviral Therapy, or Both for COVID-19: A Systematic Review.. Drugs. 2020; 80(18):1929–1946. doi: 10.1007/s40265-020-01421-w 33068263 PMC7568461

[13] Group RC, Horby P, Lim WS, Emberson JR, Mafham M, Bell JL, et al. Dexamethasone in Hospitalized Patients with Covid-19. N Engl J Med. 2021; 384(8):693–704. doi: 10.1056/NEJMoa2021436 32678530 PMC7383595

[14] Hennigan S, Kavanaugh A. Interleukin-6 inhibitors in the treatment of rheumatoid arthritis. Ther Clin Risk Manag. 2008; 4(4):767–775. doi: 10.2147/tcrm.s3470 19209259 PMC2621374

[15] Sheppard M, Laskou F, Stapleton PP, Hadavi S, Dasgupta B. Tocilizumab (Actemra). Human vaccines &*amp*; immunotherapeutics. 2017; 13(9):1972–1988. doi: 10.1080/21645515.2017.1316909 28841363 PMC5612212

[16] Pelaia C, Tinello C, Vatrella A, De Sarro G, Pelaia G. Lung under attack by COVID-19-induced cytokine storm: pathogenic mechanisms and therapeutic implications. Ther Adv Respir Dis. 2020; 14:1753466620933508. doi: 10.1177/1753466620933508 32539627 PMC7298425

[17] Rubbert-Roth A, Furst DE, Nebesky JM, Jin A, Berber E. A Review of Recent Advances Using Tocilizumab in the Treatment of Rheumatic Diseases. Rheumatol Ther. 2018; 5(1):21–42. doi: 10.1007/s40744-018-0102-x 29502236 PMC5935615

[18] Si S, Teachey DT. Spotlight on Tocilizumab in the Treatment of CAR-T-Cell-Induced Cytokine Release Syndrome: Clinical Evidence to Date. Ther Clin Risk Manag. 2020; 16:705–714. doi: 10.2147/TCRM.S223468 32801727 PMC7414980

[19] Guaraldi G, Meschiari M, Cozzi-Lepri A, Milic J, Tonelli R, Menozzi M, et al. Tocilizumab in patients with severe COVID-19: a retrospective cohort study. Lancet Rheumatol. 2020; 2(8):e474–e84. doi: 10.1016/S2665-9913(20)30173-9 32835257 PMC7314456

[20] Gupta S, Padappayil RP, Bansal A, Daouk S, Brown B. Tocilizumab in patients hospitalized with COVID-19 pneumonia: systematic review and meta-analysis of randomized controlled trials. J Investig Med. 2022; 70(1):55–60. doi: 10.1136/jim-2021-002001 34561232 PMC8476233

[21] Tantibanchachai L. FDA NEWS RELEASE, 2021 June 24 [cited 29 June 2022]. In: FDA Newsroom/ Press Announcements [Internet]. Available from: https://www.fda.gov/news-events/press-announcements/coronavirus-covid-19-update-fda-authorizes-drug-treatment-covid-19.

[22] Puig L, Bakulev AL, Kokhan MM, Samtsov AV, Khairutdinov VR, Morozova MA, et al. Efficacy and Safety of Netakimab, A Novel Anti-IL-17 Monoclonal Antibody, in Patients with Moderate to Severe Plaque Psoriasis. Results of A 54-Week Randomized Double-Blind Placebo-Controlled PLANETA Clinical Trial. Dermatol Ther (Heidelb). 2021; 11(4):1319–1332. doi: 10.1007/s13555-021-00554-4 34060012 PMC8322379

[23] Mazurov VI, Erdes SF, Gaydukova IZ, Dubinina TV, Pristrom AM, Kunder EV, et al. Long-term efficacy and safety of netakimab in the treatment of ankylosing spondylitis: results of Phase III international, multicenter, randomized double-blind clinical trial BCD-085-5/ASTERA. Modern Rheumatology Journal. 2020; 14(4):39–49. doi: 10.14412/1996-7012-2020-4-39-49

[24] Korotaeva TV, Mazurov VI, Lila AM, Gaydukova IZ, Bakulev AL, Samtsov AV, et al. Efficacy and safety of netakimab in patients with psoriatic arthritis: results of the phase III PATERA clinical study. Rheumatology Science and Practice. 2020;58. doi: 10.47360/1995-4484-2020-480-488

[25] Cua DJ, Tato CM. Innate IL-17-producing cells: the sentinels of the immune system. Nat Rev Immunol. 2010; 10(7):479–489. doi: 10.1038/nri2800 20559326

[26] Wu D, Yang XO. TH17 responses in cytokine storm of COVID-19: An emerging target of JAK2 inhibitor Fedratinib. J Microbiol Immunol Infect. 2020; 53(3):368–370. doi: 10.1016/j.jmii.2020.03.005 32205092 PMC7156211

[27] Ogura H, Murakami M, Okuyama Y, Tsuruoka M, Kitabayashi C, Kanamoto M, et al. Interleukin-17 promotes autoimmunity by triggering a positive-feedback loop via interleukin-6 induction. Immunity. 2008;29(4):628–636. doi: 10.1016/j.immuni.2008.07.018 18848474

[28] Mahallawi WH, Khabour OF, Zhang Q, Makhdoum HM, Suliman BA. MERS-CoV infection in humans is associated with a pro-inflammatory Th1 and Th17 cytokine profile. Cytokine. 2018; 104:8–13. doi: 10.1016/j.cyto.2018.01.025 29414327 PMC7129230

[29] Huang C, Wang Y, Li X, Ren L, Zhao J, Hu Y, et al. Clinical features of patients infected with 2019 novel coronavirus in Wuhan, China.. Lancet. 2020; 395(10223):497–506. doi: 10.1016/S0140-6736(20)30183-5 31986264 PMC7159299

[30] Xu Z, Shi L, Wang Y, Zhang J, Huang L, Zhang C, et al. Pathological findings of COVID-19 associated with acute respiratory distress syndrome. Lancet Respir Med. 2020;8(4):420–422. doi: 10.1016/S2213-2600(20)30076-X 32085846 PMC7164771

[31] Sara De Biasi MM, Lara Gibellini et al. Marked T cell activation, senescence, exhaustion and skewing towards TH17 in patients with Covid-19 pneumonia. Nat Commun. 2020; 11(1):3434. doi: 10.1038/s41467-020-17292-4 32632085 PMC7338513

[32] Martonik D, Parfieniuk-Kowerda A, Rogalska M, Flisiak R. The Role of Th17 Response in COVID-19. Cells. 2021; 10(6). doi: 10.3390/cells10061550 34205262 PMC8235311

[33] Ding Q, Liu GQ, Zeng YY, Zhu JJ, Liu ZY, Zhang X, et al. Role of IL-17 in LPS-induced acute lung injury: an in vivo study. Oncotarget. 2017; 8(55):93704–93711. doi: 10.18632/oncotarget.21474 29212183 PMC5706829

[34] Mikacenic C, Hansen EE, Radella F, Gharib SA, Stapleton RD, Wurfel MM. Interleukin-17A Is Associated With Alveolar Inflammation and Poor Outcomes in Acute Respiratory Distress Syndrome. Critical care medicine. 2016; 44(3):496–502. doi: 10.1097/CCM.0000000000001409 26540401 PMC4764422

[35] Li JT, Melton AC, Su G, Hamm DE, LaFemina M, Howard J, et al. Unexpected Role for Adaptive alphabetaTh17 Cells in Acute Respiratory Distress Syndrome. J Immunol. 2015; 195(1):87–95. doi: 10.4049/jimmunol.1500054 26002979 PMC4475475

[36] Szabo PA, Goswami A, Mazzuca DM, Kim K, O’Gorman DB, Hess DA, et al. Rapid and Rigorous IL-17A Production by a Distinct Subpopulation of Effector Memory T Lymphocytes Constitutes a Novel Mechanism of Toxic Shock Syndrome Immunopathology. J Immunol. 2017; 198(7):2805–2818. doi: 10.4049/jimmunol.1601366 28219889 PMC6635948

[37] Zhou X, Loomis-King H, Gurczynski SJ, Wilke CA, Konopka KE, Ptaschinski C, et al. Bone marrow transplantation alters lung antigen-presenting cells to promote TH17 response and the development of pneumonitis and fibrosis following gammaherpesvirus infection. Mucosal immunology. 2016; 9(3):610–620. doi: 10.1038/mi.2015.85 26376362 PMC4794430

[38] Guabiraba R, Besnard AG, Marques RE, Maillet I, Fagundes CT, Conceicao TM, et al. IL-22 modulates IL-17A production and controls inflammation and tissue damage in experimental dengue infection. Eur J Immunol. 2013; 43(6):1529–1544 doi: 10.1002/eji.201243229 23505056

[39] Xie M, Cheng B, Ding Y, Wang C, Chen J. Correlations of IL-17 and NF-kappaB gene polymorphisms with susceptibility and prognosis in acute respiratory distress syndrome in a chinese population. Biosci Rep. 2019; 39(2). doi: 10.1042/BSR20181987 30655311 PMC6367126

[40] Zhang X, Zhang Y, Qiao W, Zhang J, Qi Z. Baricitinib, a drug with potential effect to prevent SARS-COV-2 from entering target cells and control cytokine storm induced by COVID-19. International immunopharmacology. 2020; 86:106749. doi: 10.1016/j.intimp.2020.106749 32645632 PMC7328558

[41] Kubo S, Nakayamada S, Sakata K, Kitanaga Y, Ma X, Lee S, et al. Janus Kinase Inhibitor Baricitinib Modulates Human Innate and Adaptive Immune System. Frontiers in immunology. 2018; 9:1510. doi: 10.3389/fimmu.2018.01510 30002661 PMC6031708

[42] Jorgensen SCJ, Tse CLY, Burry L, Dresser LD. A Review of Pharmacology, Safety, and Emerging Clinical Experience in COVID-19. Pharmacotherapy. 2020;4 0(8):843–856. doi: 10.1002/phar.2438 32542785 PMC7323235

[43] Marconi VC, Ramanan AV, de Bono S, Kartman CE, Krishnan V, Liao R, et al. EEfficacy and safety of baricitinib for the treatment of hospitalised adults with COVID-19 (COV-BARRIER): a randomised, double-blind, parallel-group, placebo-controlled phase 3 trial. Lancet Respir Med. 2021; 9(12):1407–1418. doi: 10.1016/S2213-2600(21)00331-3 34480861 PMC8409066

[44] Lan SH, Wang CK, Chang SP, Lu LC, Hung SH, Lin WT. Janus kinase inhibitors for hospitalized patients with COVID-19: a meta-analysis of randomized controlled trials. Expert Rev Anti Infect Ther. 2021:1–7. doi: 10.1080/14787210.2022.2004120 34743661 PMC8607541

[45] Smith GB, Redfern OC, Pimentel MA, Gerry S, Collins GS, Malycha J, et al. The National Early Warning Score 2 (NEWS2). Clin Med (Lond). 2019; 19(3):260. doi: 10.7861/clinmedicine.19-3-260 31092526 PMC6542226

[46] Myrstad M, Ihle-Hansen H, Tveita AA, Andersen EL, Nygard S, Tveit A, et al. National Early Warning Score 2 (NEWS2) on admission predicts severe disease and in-hospital mortality from Covid-19—a prospective cohort study. Scand J Trauma Resusc Emerg Med. 2020; 28(1):66. doi: 10.1186/s13049-020-00764-3 32660623 PMC7356106

[47] Kostakis I, Smith GB, Prytherch D, Meredith P, Price C, Chauhan A, et al. The performance of the National Early Warning Score and National Early Warning Score 2 in hospitalised patients infected by the severe acute respiratory syndrome coronavirus 2 (SARS-CoV-2). Resuscitation. 2021; 159:150–7. doi: 10.1016/j.resuscitation.2020.10.039 33176170 PMC7648887

[48] Ministry of Health of Russian Federation, Interim Guidelines. The prevention, diagnosis and treatment of novel coronavirus infection (COVID-19), version 4 (03/27/2020) [Cited 2022 June 29]. In Minzdrav.gov.ru [Internet]. Available from: https://static-0.minzdrav.gov.ru/system/attachments/attaches/000/049/881/original/COVID19_recomend_v4.pdf

[49] Martins-Filho PR, Ferreira LC, Heimfarth L, Araujo AAS, Quintans-Junior LJ. Efficacy and safety of hydroxychloroquine as pre-and post-exposure prophylaxis and treatment of COVID-19: A systematic review and meta-analysis of blinded, placebo-controlled, randomized clinical trials. Lancet Reg Health Am. 2021; 2:100062. doi: 10.1016/j.lana.2021.100062 34485970 PMC8403035

[50] WHO Media inquiries News release, 2021 July 6 [cited 29 June 2022]. In: WHO/News [Internet], Geneva. Available from: https://www.who.int/news/item/06-07-2021-who-recommends-life-saving-interleukin-6-receptor-blockers-for-covid-19-and-urges-producers-to-join-efforts-to-rapidly-increase-access

[51] Hermine O, Mariette X, Tharaux PL, Resche-Rigon M, Porcher R, Ravaud P, et al. Effect of Tocilizumab vs Usual Care in Adults Hospitalized With COVID-19 and Moderate or Severe Pneumonia: A Randomized Clinical Trial. JAMA Intern Med. 2021; 181(1):32–40. doi: 10.1001/jamainternmed.2020.6820 33080017 PMC7577198

[52] Samaee H, Mohsenzadegan M, Ala S, Maroufi SS, Moradimajd P. Tocilizumab for treatment patients with COVID-19: Recommended medication for novel disease. International immunopharmacology. 2020; 89(Pt A):107018. doi: 10.1016/j.intimp.2020.107018 33045577 PMC7494278

[53] Stone JH, Frigault MJ, Serling-Boyd NJ, Fernandes AD, Harvey L, Foulkes AS, et al. Efficacy of Tocilizumab in Patients Hospitalized with Covid-19. N Engl J Med. 2020; 383(24):2333–2344 doi: 10.1056/NEJMoa2028836 33085857 PMC7646626

[54] Rosas IO, Brau N, Waters M, Go RC, Hunter BD, Bhagani S, et al. Tocilizumab in Hospitalized Patients with Severe Covid-19 Pneumonia. N Engl J Med. 2021; 384(16):1503–1516. doi: 10.1056/NEJMoa2028700 33631066 PMC7953459

[55] Gatti M, Fusaroli M, Caraceni P, Poluzzi E, De Ponti F, Raschi E. Serious adverse events with tocilizumab: Pharmacovigilance as an aid to prioritize monitoring in COVID-19. British journal of clinical pharmacology. 2021; 87(3):1533–1540. doi: 10.1111/bcp.14459 32639062

[56] Charan J, Dutta S, Kaur R, Bhardwaj P, Sharma P, Ambwani S, et al. Tocilizumab in COVID-19: a study of adverse drug events reported in the WHO database. Expert Opin Drug Saf. 2021; 20(9):1125–1136. doi: 10.1080/14740338.2021.1946513 34162299 PMC8290369

[57] Pettit NN, Nguyen CT, Mutlu GM, Wu D, Kimmig L, Pitrak D, et al. Late onset infectious complications and safety of tocilizumab in the management of COVID-19. J Med Virol. 2021; 93(3):1459–1464. doi: 10.1002/jmv.26429 32790075 PMC7436682

[58] Shi JG, Chen X, Lee F, Emm T, Scherle PA, Lo Y, et al. The pharmacokinetics, pharmacodynamics, and safety of baricitinib, an oral JAK 1/2 inhibitor, in healthy volunteers. J Clin Pharmacol. 2014; 54(12):1354–1361. doi: 10.1002/jcph.354 24965573

[59] Keystone EC, Genovese MC, Schlichting DE, de la Torre I, Beattie SD, Rooney TP, et al. Safety and Efficacy of Baricitinib Through 128 Weeks in an Open-label, Longterm Extension Study in Patients with Rheumatoid Arthritis. J Rheumatol. 2018; 45(1):14–21. doi: 10.3899/jrheum.161161 28811354

[60] Stebbing J, Sanchez Nievas G, Falcone M, Youhanna S, Richardson P, Ottaviani S, et al. JAK inhibition reduces SARS-CoV-2 liver infectivity and modulates inflammatory responses to reduce morbidity and mortality. Science advances. 2021; 7(1). doi: 10.1126/sciadv.abe4724 33187978 PMC7775747

[61] Maslennikov R, Ivashkin V, Vasilieva E, Chipurik M, Semikova P, Semenets V, et al. Interleukin 17 antagonist netakimab is effective and safe in the new coronavirus infection (COVID-19). Eur Cytokine Netw. 2021; 32(1):8–14. doi: 10.1684/ecn.2021.0463 34346869 PMC8491178

[62] Avdeev SN, Trushenko NV, Tsareva NA, Yaroshetskiy AI, Merzhoeva ZM, Nuralieva GS, et al. AAnti-IL-17 monoclonal antibodies in hospitalized patients with severe COVID-19: A pilot study. Cytokine. 2021; 146:155627. doi: 10.1016/j.cyto.2021.155627 34237556 PMC8253694

[63] Resende GG, da Cruz Lage R, Lobe SQ, Medeiros AF, Costa ESAD, Nogueira Sa AT, et al. Blockade of interleukin seventeen (IL-17A) with secukinumab in hospitalized COVID-19 patients—the BISHOP study. Infect Dis (Lond). 2022; 54(8):591–599. doi: 10.1080/23744235.2022.2066171 35485381

[64] Yin Y, Wang M, Liu M, Zhou E, Ren T, Chang X, et al. Efficacy and safety of IL-17 inhibitors for the treatment of ankylosing spondylitis: a systematic review and meta-analysis. Arthritis Res Ther. 2020;22(1):111. doi: 10.1186/s13075-020-02208-w 32398096 PMC7216398

