# Supplementary figures and images for: Tocilizumab, netakimab, and baricitinib in patients with mild-to-moderate COVID-19: An observational study
Source: PLoS One. 2022 Aug 24;17(8):e0273340. doi: 10.1371/journal.pone.0273340 (PMC9401152; doi:10.1371/journal.pone.0273340)

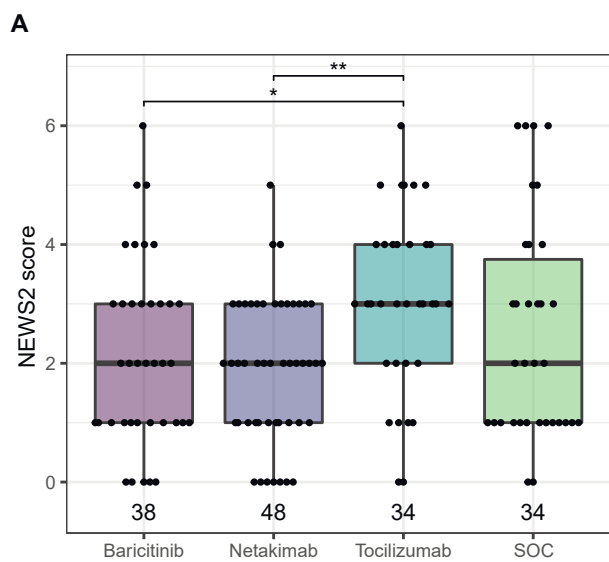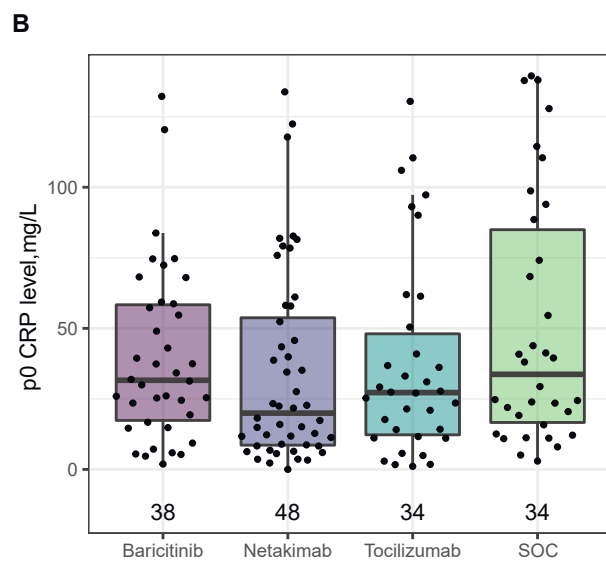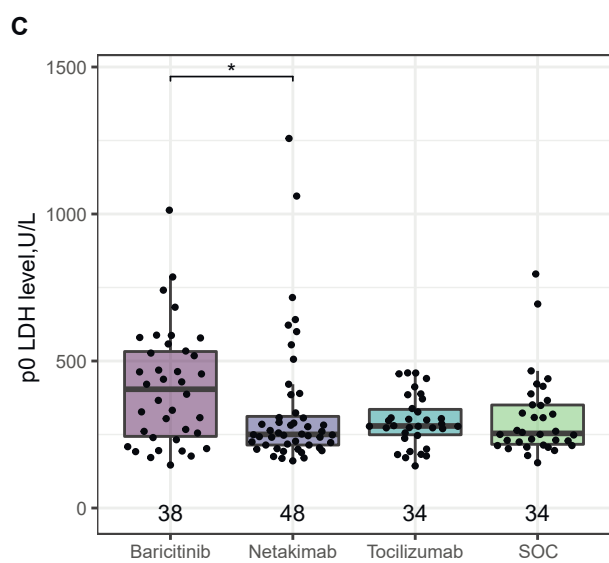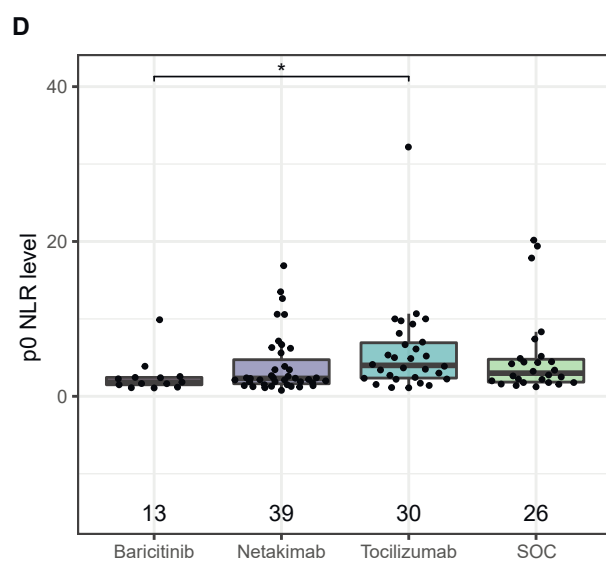

Supplement: S1 Fig — Kruskal-Wallis and Dunn test, Bonferroni adjustment method: *p < 0.05; **p < 0.01; ***p < 0.001. Number of patients with available data are shown along the x-axis. (PDF) [file pone.0273340.s001.pdf]

**A**

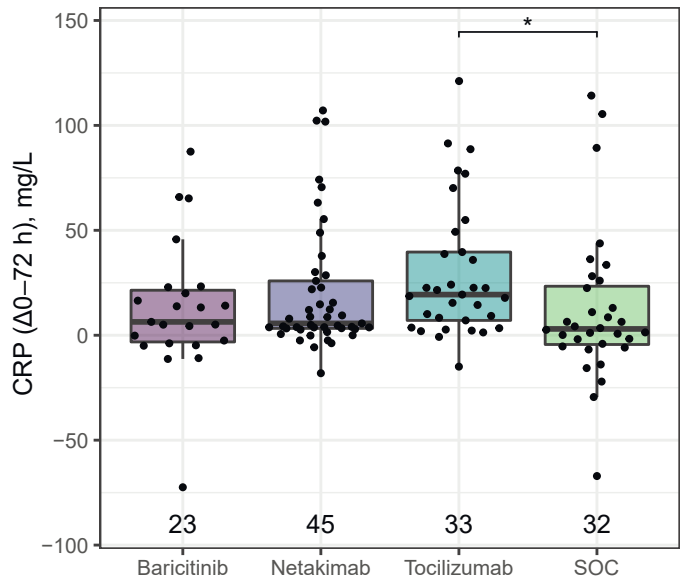

**B**

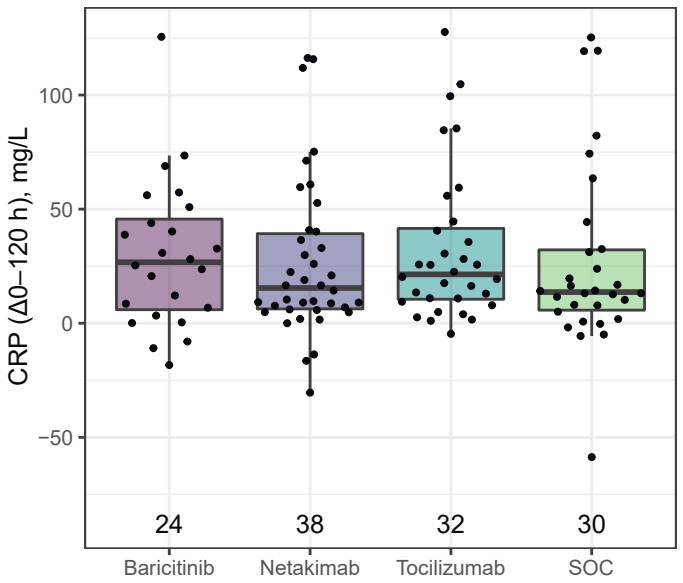

**C**

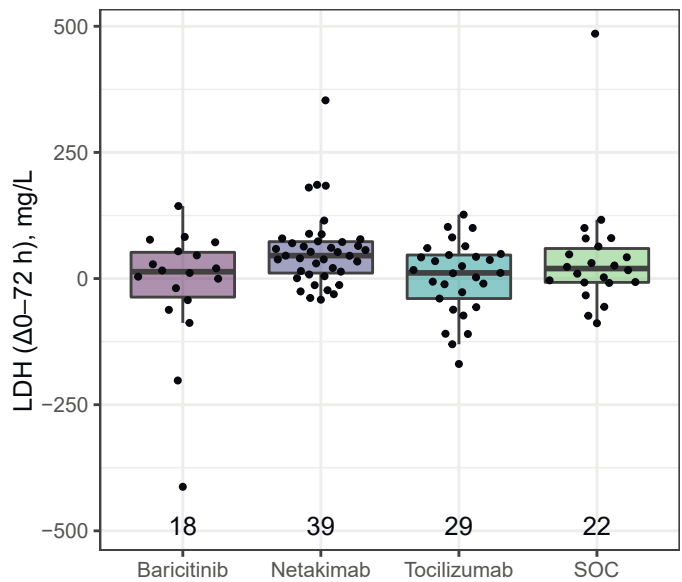

**D**

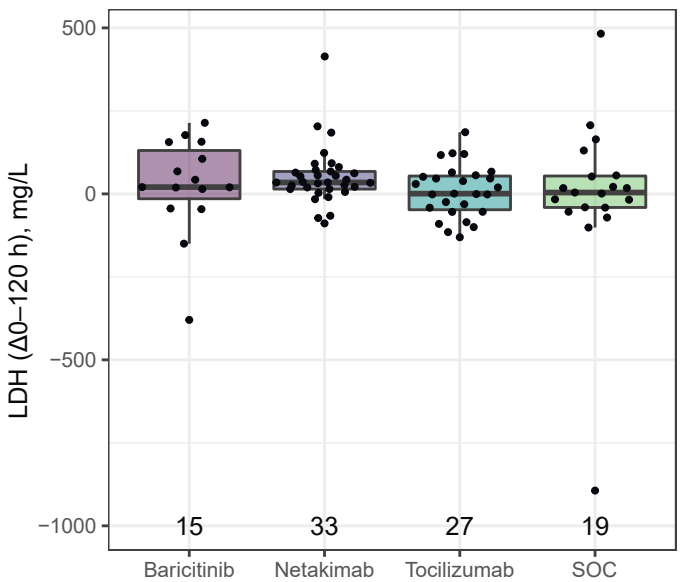

Supplement: S2 Fig — Kruskal-Wallis and Dunn test: *p < 0.05. Number of patients with available data are shown along the x-axis. (PDF) [file pone.0273340.s002.pdf]

**a**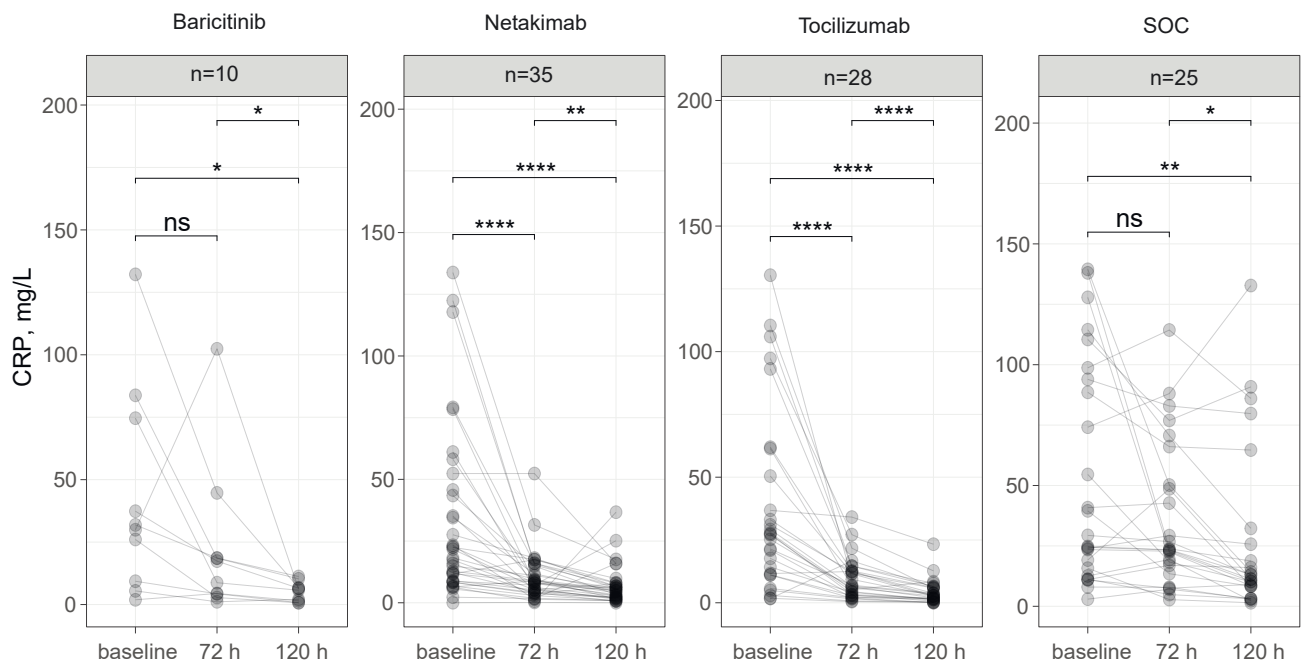**b**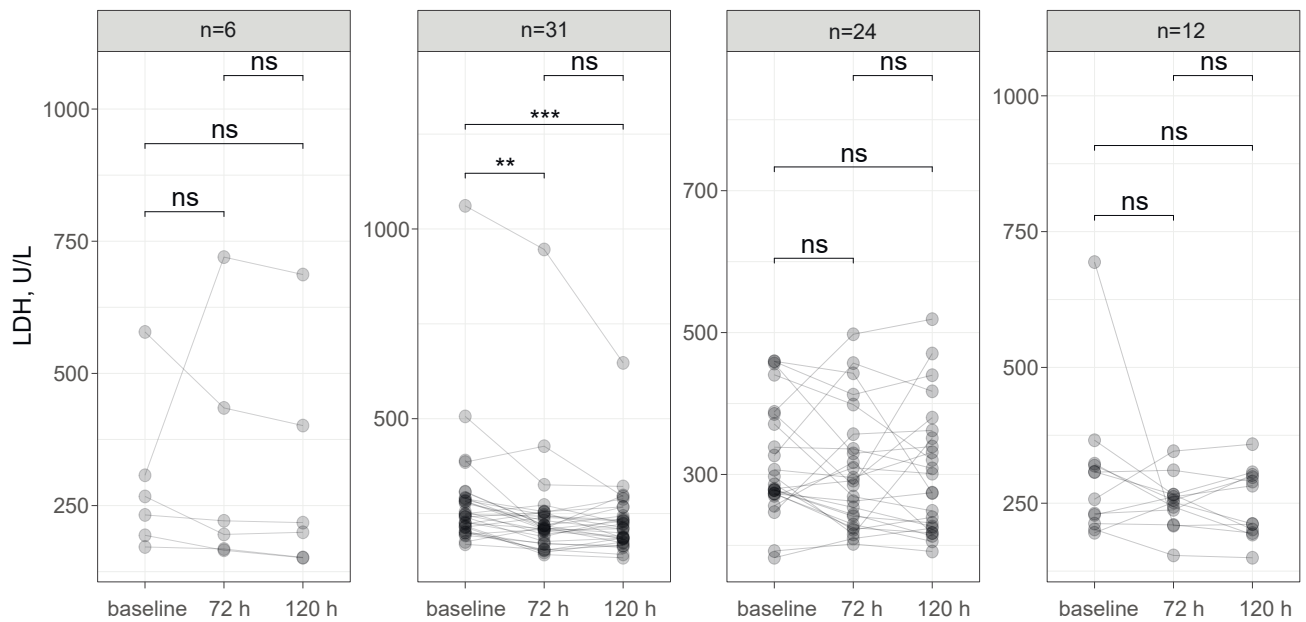

Supplement: S3 Fig — Wilcoxon test: *p < 0.05; **p < 0.01; ***p < 0.001; ****p < 0.0001, ns = non-significant; n = number of patients with available data for all 3 time-points. (PDF) [file pone.0273340.s003.pdf]
