# Supplementary material for: Tocilizumab, netakimab, and baricitinib in patients with mild-to-moderate COVID-19: An observational study
Source: PLoS One. 2022 Aug 24;17(8):e0273340. doi: 10.1371/journal.pone.0273340 (PMC9401152; doi:10.1371/journal.pone.0273340)
Supplement: S1 Table — Data are median (IQR), where IQR = interquartile range; CRP = C-reactive protein (mg/L); LDH = lactic dehydrogenase (IU/L); ANC = absolute neutrophil count (x 109/L); ALC = absolute lymphocyte count (x 109/L); NLR = neutrophil-to-lymphocyte ratio; NEWS2 = National Early Warning Score 2; n = number of patients with corresponding data. P values were calculated using Kruskal-Wallis and Dunn test, Δ = p-value for Baricitinib group, ‡ = p-value for Netakimab group, # = p-value for Tocilizumab group, ⬛ = p-value for SOC group. P-values that do not significantly differ are shown as p > 0.05. (PDF) [file pone.0273340.s004.pdf]

S1 Table. Clinical characteristics of patients from the Δ0–72 h and Δ0–120 h groups.

	**Baricitinib**	**Netakimab**	**Tocilizumab**	**SOC**	**p-value**
0-72 hours					
n, CRP	24	45	33	32	
CRP 0 h median (IQR)	28 (13.28–57.65)	17.34 (8.32–52.36)	27.39 (14.09–50.44)	27.05 (14.98–89.91)	>0.05 △<br>8 x 10^{-4} ‡<br>1 x 10^{-5} #<br>>0.05 ■
CRP 72 h median (IQR)	17.09 (7.93–25.76)	**7.89 (4.09–14.87)**	**6.6 (2.81–12.67)**	23.34 (13.47–54.16)	
n, LDH	18	39	29	22	
LDH 0 h median (IQR)	327 (220.45–491.5)	247.6 (209.45–298.8)	286 (272.6–371.1)	282.25 (216.47–350.4)	>0.05 △<br>0.029 ‡<br>>0.05 #<br>>0.05 ■
LDH 72 h median (IQR)	332.8 (208.3–448.5)	210 (174.8–250.1)	291.3 (241–356.8)	253.65 (210.32–320.15)	
n, ANC, ALC, NLR	26	46	34	32	
ANC 0 h median (IQR)	5.05 (2.8–7.06)	3.05 (1.92–4.95)	3.25 (2.25–4.47)	4.2 (2.85–6.47)	>0.05 △<br>0.0006408 ‡<br>0.02806 #<br>>0.05 ■
ANC 72 h median (IQR)	3.21 (2.05–5.41)	**5.4 (3.05–8.27)**	**2 (1.42–3.87)**	3.9 (2.7–4.77)	
ALC 0 h median (IQR)	1.65 (1.08–2,.7)	1 (0.72–1.6)	1.05 (0.62–1.37)	1.2 (0.87–1.52)	0.01274 △<br>>0.05 ‡<br>>0.05 #<br>>0.05 ■
ALC 72 h median (IQR)	**2.65 (1.9–3.39)**	1.15 (0.75–1.4)	1.1 (0.8–1.4)	1.3 (0.97–1.62)	
NLR 0 h median (IQR)	2.41 (1.64–4.11)	2.32 (1.77–5.46)	3.93 (2.35–6.52)	3.3 (1.94–5.64)	0.009367△<br>0.00608 ‡<br>0.01416 #<br>>0.05 ■
NLR 72 h median (IQR)	**1.18 (0.8–1.68)**	**5.26 (2.36–7.59)**	2.05 (0.94–5)	2.75 (1.56–4.8)	
n, NEWS2	38	48	34	34	
NEWS2 0 h median (IQR)	2 (1–3)	2 (1–3)	3 (2–4)	2 (1–3.75)	0.0432 △<br>0.0104 ‡<br>0.0000684 #<br>>0.05 ■
NEWS2 72 h median (IQR)	1 (0–2.75)	1 (0–2)	2 (1–2)	1 (1–2)	

0–120 hours					
n, CRP	23	38	32	30	
CRP 0 h median (IQR)	31.89 (21.45–51.85)	19.95 (8.69–45.14)	27.22 (11.52–43.3)	34.43 (13.36–92.59)	1.2×10^{-4} △ 6.3×10^{-7} ‡ 1.9×10^{-7} # 0.003 ■
CRP 120 h median (IQR)	**6.7 (3.71–11.06)**	**4.84 (1.99–7.85)**	**2.64 (1.11–5.70)**	**12.13 (8.5–25.38)**	
n, LDH	15	33	25	19	
LDH 0 h median (IQR)	332.5 (249.8–523.7)	247.6 (214.3–291.2)	286 (272.8–371.1)	257.3 (209.8–321)	>0.05 △ >0.05 ‡ >0.05 # >0.05 ■
LDH 120 h median (IQR)	401.3 (208.6–526.5)	212.9 (184.9–267.3)	274.4 (226.6–339.4)	246.8 (204.35–293.9)	
n, ANC, ALC, NLR	19	38	31	28	
ANC 0 h median (IQR)	3.9 (2.5–6.34)	3 (1.95–4.35)	3.2 (2.25–4.45)	3.95 (2.7–5.52)	>0.05 △ 6.9×10^{-6} ‡ >0.05 # >0.05 ■
ANC 120 h median (IQR)	3.37 (2.65–4.18)	**6.2 (4.6–7.55)**	3.4 (2.25–4.5)	3.95 (2.27–5.22)	
ALC 0 h median (IQR)	1.6 (1.14–2.59)	1 (0.7–1.6)	1 (0.6–1.4)	1.2 (0.9–1.52)	0.03108 △ >0.05 ‡ >0.05 # >0.05 ■
ALC 120 h median (IQR)	**2.4 (2.05–3.47)**	1.2 (1–1.6)	1.3 (0.8–1.81)	1.2 (0.9–1.8)	
NLR 0 h median (IQR)	2.25 (1.56–3.21)	2.31 (1.62–4.73)	4.09 (2.38–6.83)	2.99 (1.94–4.94)	4.37×10^{-5} △ 0.008684 ‡ >0.05 # >0.05 ■
NLR 120 h median (IQR)	**1.36 (0.91–1.88)**	**5.13 (2.57–6.98)**	2.79 (1.34–6.2)	2.19 (1.83–5.53)	
n, NEWS	38	48	34	34	
NEWS2 0 h median (IQR)	2 (1–3)	2 (1–3)	3 (2–4)	2 (1–3.75)	0.00046 △ 0.00102 ‡ 0.000086 # >0.05 ■
NEWS2 120 h median (IQR)	1 (0–1)	1 (0–2)	1 (0–2)	1 (1–3)	

Data are median (IQR), where IQR = interquartile range; CRP = C-reactive protein (mg/L); LDH = lactic dehydrogenase (IU/L); ANC = absolute neutrophil count (x 10^9/L); ALC = absolute lymphocyte count (x 10^9/L); NLR = neutrophil-to-lymphocyte ratio; NEWS2 = National Early Warning Score 2; n = number of patients with corresponding data. P values were calculated using Kruskal-Wallis and Dunn test, △ = p-value for Baricitinib group, ‡ = p-value for Netakimab group, # = p-value for Tocilizumab group, ■ = p-value for SOC group. P-values that do not significantly differ are shown as p >0.05.
