# Supplementary material for: Tocilizumab, netakimab, and baricitinib in patients with mild-to-moderate COVID-19: An observational study
Source: PLoS One. 2022 Aug 24;17(8):e0273340. doi: 10.1371/journal.pone.0273340 (PMC9401152; doi:10.1371/journal.pone.0273340)
Supplement: S2 Table — Data are median (IQR), where IQR = interquartile range; CRP = C-reactive protein (mg/L); LDH = lactic dehydrogenase (IU/L); ANC = absolute neutrophil count (x 109/L); ALC = absolute lymphocyte count (x 109/L); NLR = neutrophil-to-lymphocyte ratio; n = number of patients with data available. (PDF) [file pone.0273340.s005.pdf]

S2 Table. Clinical characteristics of patients at three different timepoints.

	Baricitinib	Netakimab	Tocilizumab	SOC
n, CRP	10	35	28	25
CRP 0 h median (IQR)	31.89 (14.6–72.4)	17.76 (8.59–44.01)	27.39 (12.87–45.68)	27 (14.98–95.13)
CRP 72 h median (IQR)	18,46 (4.34–37.93)	**7.9 (3.98–12.55)**	**6.48 (2.52–12.4)**	23.6 (16.09–67.23)
CRP 120 h median (IQR)	5.73 (2.1–6.7)	**4.72 (1.96–6.95)**	**2.7 (0.98–5.75)**	12.13 (8.44–27.34)
n, LDH	6	31	24	12
LDH 0 h median (IQR)	267.3 (213.05–319.9)	245.9 (214.25–286.8)	280 (272.6–371.1)	257.3 (212.5–319.2)
LDH 72 h median (IQR)	221.2 (181.65–383.75)	211.6 (182.7–244.9)	285 (238.5–336)	251.6 (120.1–265.7)
LDH 120 h median (IQR)	217.7 (175.7–314.15)	210.1 (183.95–240.25)	274,4 (226.6–351.2)	248,6 (201.6–298)
n, ANC, ALC, NLR	13	39	30	26
ANC 0 h median (IQR)	2.8 (2.2–3.9)	3 (1.95–4.35)	3.4 (2.22–4.3)	3.95 (2.7–5.97)
ANC 72 h median (IQR)	2.2 (1.8–2.9)	**5.3 (2.9–8.4)**	2.1 (1.35–4.12)	3.65 (2.62–4.82)
ANC 120 h median (IQR)	2.9 (2.2–3.37)	**6.2 (4.6–7.55)**	3.45 (2.47–4.7)	3.65 (2.22–5.67)
ALC 0 h median (IQR)	1.5 (0.92–1.9)	1 (0.7-1.5)	0.95 (0.6–1.4)	1.2 (0.9–1.57)
ALC 72 h median (IQR)	**2.3 (1.9–3.2)**	1.2 (0.75–1.4)	1.05 (0.72–1.3)	1.35 (1–1.9)
ALC 120 h median (IQR)	**2.2 (2–2.4)**	1.2 (1–1.6)	1.3 (0.8–1.8)	1.15 (0.9–1.75)
NLR 0 h median (IQR)	2.4 (1.48–3.87)	2.32 (1.58–3.75)	3.99 (2.35–6.91)	2.56 (1.77–6.29)
NLR 72 h median (IQR)	1.31 (0.96–2.44)	**4.85 (2.44–8.12)**	2.81 (1.04–5.04)	2.75 (1.63–6.75)
NLR 120 h median (IQR)	1.57 (1.16–2.07)	**5.2 (2.26–7.07)**	2.89 (1.38–6.3)	3.41 (1.92–5.9)

Data are median (IQR), where IQR = interquartile range; CRP = C-reactive protein (mg/L); LDH = lactic dehydrogenase (IU/L); ANC = absolute neutrophil count (x 10^9/L); ALC = absolute lymphocyte count (x 10^9/L); NLR = neutrophil-to-lymphocyte ratio; n = number of patients with data available.
